# Association of mild and complex multimorbidity with structural brain changes in older adults: A population‐based study

**DOI:** 10.1002/alz.13614

**Published:** 2024-01-03

**Authors:** Martina Valletta, Davide Liborio Vetrano, Amaia Calderón‐Larrañaga, Grégoria Kalpouzos, Marco Canevelli, Alessandra Marengoni, Erika J Laukka, Giulia Grande

**Affiliations:** ^1^ Aging Research Center Department of Neurobiology Care Sciences and Society Karolinska Institutet and Stockholm University Stockholm Sweden; ^2^ Stockholm Gerontology Research Center Stockholm Sweden; ^3^ Department of Human Neuroscience Sapienza University Rome Italy; ^4^ Department of Clinical and Experimental Sciences University of Brescia Brescia Italy

**Keywords:** brain changes, brain magnetic resonance imaging, cognitive decline, multimorbidity, neuroimaging, population‐based study

## Abstract

**INTRODUCTION:**

We quantified the association of mild (ie, involving one or two body systems) and complex (ie, involving ≥3 systems) multimorbidity with structural brain changes in older adults.

**METHODS:**

We included 390 dementia‐free participants aged 60+ from the Swedish National Study on Aging and Care in Kungsholmen who underwent brain magnetic resonance imaging at baseline and after 3 and/or 6 years. Using linear mixed models, we estimated the association between multimorbidity and changes in total brain tissue, ventricular, hippocampal, and white matter hyperintensities volumes.

**RESULTS:**

Compared to non‐multimorbid participants, those with complex multimorbidity showed the steepest reduction in total brain (β*time −0.03, 95% CI −0.05, −0.01) and hippocampal (β*time −0.05, 95% CI −0.08, −0.03) volumes, the greatest ventricular enlargement (β*time 0.03, 95% CI 0.01, 0.05), and the fastest white matter hyperintensities accumulation (β*time 0.04, 95% CI 0.01, 0.07).

**DISCUSSION:**

Multimorbidity, particularly when involving multiple body systems, is associated with accelerated structural brain changes, involving both neurodegeneration and vascular pathology.

**Highlights:**

- Multimorbidity accelerates structural brain changes in cognitively intact older adults
- These brain changes encompass both neurodegeneration and cerebrovascular pathology
- The complexity of multimorbidity is associated with the rate of brain changes’ progression

## 1 BACKGROUND

Several chronic diseases, including but not limited to atrial fibrillation,^1^ diabetes^2^ and pulmonary diseases,^3^ have long been recognized for their association with changes in brain structure and function. These conditions contribute to global brain atrophy, hippocampal shrinkage, and the accumulation of white matter lesions,^1^, ^2^, ^3^ and have been linked to different cognitive decline trajectories. However, it is rare for older adults to suffer from a single chronic disease. Multimorbidity, which refers to the co‐occurrence of two or more chronic diseases within the same individual, becomes increasingly prevalent after the age of 70.^4^ The co‐occurrence of different diseases in an individual can be attributed to shared risk factors and common underlying pathophysiological mechanisms, and a growing body of evidence highlights the impact of disease combinations—or disease‐clusters—on various negative health outcomes.^5^ Cognitive impairment,^6^, ^7^ dementia,^8^, ^9^, ^10^ and reduced survival^11^ have all been linked to multimorbidity and different disease clusters.

Recent findings suggest that when diseases affecting different body systems, such as the cardiocirculatory, metabolic, and respiratory systems, coexist within the same individual, their impact on health outcomes exceeds the mere sum of the individual diseases.^11^, ^12^, ^13^ In other words, the interplay between diseases affecting different body systems may amplify their effects. Whether this happens in relation to structural brain changes is unknown. Understanding the role played by multimorbidity in the occurrence as well as the progression of brain changes is essential to better elucidate the underlying pathophysiological mechanisms linking the high burden of chronic diseases often found in old age to cognitive decline and overt dementia.

The primary objective of our study was to quantify the impact of multimorbidity burden, with a focus on the number of co‐occurring diseases and impaired body systems, on structural brain changes, encompassing brain atrophy and cerebrovascular lesions load. To accomplish this, we conducted our analyses on a well‐characterized, population‐based cohort with repeated brain magnetic resonance imaging (MRI).

## 2 METHODS

### 2.1 Study population

For this study, we used data from the MRI substudy of the Swedish National study on Aging and Care in Kungsholmen (SNAC‐K‐MRI). SNAC‐K is an ongoing population study that started in 2001 with the enrolment of 3363 individuals aged 60 years or older (73% participation rate). The SNAC‐K study design has been described previously.^14^

**RESEARCH IN CONTEXT**

1. **Systematic review**: The authors reviewed the literature (PubMed and Embase) and found only a few cross‐sectional studies investigating the association of multimorbidity with structural brain changes.
2. **Interpretation**: Multimorbidity accelerates structural brain changes in older adults, involving neurodegeneration and vascular pathology. There is a dose‐response relationship between the complexity of multimorbidity and the rate of progression of brain atrophy and cerebrovascular lesions’ load. These results were observed among cognitively intact individuals, suggesting that multimorbidity burden has an early detrimental effect on brain.
3. **Future directions**: Further studies are warranted to elucidate the biological mechanisms that underlie the relationship between multimorbidity and dementia. Chronic disease burden should be a target for preventive strategies with the aim to preserve brain health.

Among SNAC‐K participants, a subgroup of 555 dementia‐free, non‐disabled and non‐institutionalized individuals underwent structural brain 1.5 tesla MRI scans. The scans were repeated after 6 years for the younger cohorts (ie, participants aged <78 years) and after 3 and 6 years for the older ones (ie, participants aged ≥78 years).

In order to focus on the impact of somatic diseases on brain structure, in the present study, we excluded from the subsample of individuals who underwent brain MRI those with any neuropsychiatric disease (ie, cerebrovascular diseases *n* = 21, tumors *n* = 11, migraine *n* = 11, parkinsonism *n* = 4, dementia *n* = 2, other neurological diseases *n* = 14, depression *n* = 32, other psychiatric/behavioral disturbances *n* = 17), and participants with suboptimal MRI quality (*n* = 53), leaving a final sample of 390 individuals.

The protocol for all waves of the SNAC‐K study was approved by the Regional Ethical Review Board in Stockholm. All participants provided written informed consent to participate at each study visit.

The results of this study are reported following the STROBE recommendations.

### 2.2 Data collection

At each study visit, data were collected by trained nurses and physicians following standard procedures.

Data on sociodemographic variables (ie, age, sex, and education) were obtained through face‐to‐face interviews performed by nurses. Educational attainment was categorized into three levels: elementary, high school, and university or higher. Peripheral blood samples were collected for DNA extraction and apolipoprotein E (*APOE*) genotyping. Participants were divided into *APOE‐ ε*4 carriers or non‐carriers if they had at least one *ε*4 allele or none, respectively. Global cognition was assessed using the Mini‐Mental State Examination (MMSE)^15^ and the MMSE score was categorized into ≥27 and <27.^16^ Dementia was diagnosed across all waves according to the Diagnostic and Statistical Manual of Mental Disorders, 4th edition (DSM‐IV) criteria, using a three‐step procedure.^17^ If a participant died between two visits without a dementia diagnosis, further information was obtained via (1) the clinical charts and medical records, and (2) the Swedish National Cause of Death Register.

#### 2.2.1 Chronic disease assessment

Chronic diseases were diagnosed at baseline based on participants’ medical history, medical examinations performed by physicians, participants’ and/or proxies’ interviews, diagnostic tests including instrumental and laboratory tests, and use of medications. Data from the Swedish National Patient Register including medical journals and inpatient and outpatient records were also integrated and considered. Diseases were coded according to the International Classification of Diseases 10th revision (ICD‐10) and further classified into sixty chronic diseases. The full methodology used in SNAC‐K for the detection and classification of chronic diseases has been described in detail elsewhere.^4^

In the present study, diseases diagnosed at baseline were further grouped into twelve different body systems (ie, diseases of the cardiocirculatory system; diseases of the endocrine and metabolic system; diseases of the genitourinary system; musculoskeletal conditions; diseases of the digestive system; diseases of the respiratory system; hematological and immunological conditions; diseases of the eye; cancers; diseases of the ear, nose, and throat; infectious diseases; skin conditions) following the ICD‐10 chapters and in line with previous literature.^18^ Chronic diseases and body systems considered for the definition of multimorbidity are listed in Table S1. Multimorbidity was defined as the co‐occurrence of two or more chronic diseases in the same individual and was further classified into mild (ie, the co‐occurrence of two or more chronic diseases affecting one or two body systems) or complex (ie, the co‐occurrence of three or more chronic diseases affecting three or more body systems), following a methodology previously adopted by other authors.^18^

### 2.3 Neuroimaging data acquisition and processing

All participants were scanned with a 1.5T MRI (Philips Intera, The Netherlands), and the same scanner and sequences were used for both baseline and follow‐up examinations. To calculate brain volumes, SPM12 software in MATLAB 10 was used. T1‐weighted MRI images were segmented into grey matter (GMV), white matter (WMV), and cerebrospinal fluid (CSFV) volumes. Total brain tissue volume (TBTV) was obtained by summing GMV and WMV, and total intracranial volume (TIV) was calculated as the sum of GMV, WMV, and CSFV. Hippocampal volume was calculated using FreeSurfer automated segmentation and was obtained as the sum of the volumes of the left and right hippocampus. The volumes of the lateral ventricles were calculated using automated segmentation in the ALVIN toolbox.^19^ A neuroimaging expert (GK) visually assessed all the segmentations and manually drew the white matter hyperintensities (WMH) volume on fluid‐attenuated inversion recovery (FLAIR) images and further interpolated them on the corresponding T1‐weighted images to compensate for the gap between slices in FLAIR, using MRIcron software.

The volumes of total brain tissue, hippocampus, lateral ventricles, and WMH were all adjusted for TIV. To allow comparison between coefficients, all volumes were converted into z‐scores using the mean and standard deviation of the study population at baseline.

Details of the MRI acquisition protocol and imaging processing are described in the Supplementary methods.

### 2.4 Statistical analysis

We used linear mixed models to estimate the association between the presence of multimorbidity (non‐multimorbid individuals as reference) and the number of chronic diseases (continuous measure) at baseline and the levels (intercept) and rates of change (slope) in brain volumes. Then, we used linear mixed models to estimate the association between the presence of mild and complex multimorbidity at baseline (non‐multimorbid individuals as reference) and the levels (intercept) and rates of change (slope) in brain volumes. We used linear mixed models with random participant‐specific intercepts and random slopes for follow‐up time. The interaction terms between follow‐up time and the variables related to multimorbidity were included as a fixed effect. All analyses were adjusted for potential confounders including age, sex, education, and *APOE* genotype.

Statistical analyses were performed using Stata, version 17 (StataCorp, TX).

## 3 RESULTS

At baseline, study participants had a mean (SD) age of 70.8 (8.9) years, 223 (57.7%) of them were women, 159 (40.8%) had an educational attainment of university or above, and 11 (2.8%) had an MMSE score below 27. Among the 390 participants, 306 (78.5%) were affected by multimorbidity. Among them, 106 (27.2%) had mild, while 200 (51.3%) had complex multimorbidity. Baseline characteristics of the study population are reported in Table 1.

**TABLE 1:** Baseline characteristics of the study population, overall and by presence of mild/complex multimorbidity.

	Overall (*N* = 390)	No multimorbidity (*N* = 84)	Mild multimorbidity (*N* = 106)	Complex multimorbidity (*N* = 200)
**Demographics**				
Age (years), mean (SD)	70.8 (8.9)	64.7 (6.0)	68.5 (8.0)	74.6 (8.6)
Sex (F), *n* (%)	223 (57.7)	44 (52.4)	58 (54.7)	123 (61.5)
Education (university), *n* (%)	159 (40.8)	48 (57.1)	45 (42.5)	66 (33.0)
***APOE* (*ε*4 carriers), *n* (%)**	104 (27.6)	21 (25.9)	38 (37.3)	45 (23.2)
**MMSE <27, *n* (%)**	11 (2.8)	2 (2.4)	4 (3.8)	5 (2.5)
**Chronic diseases, *n* (%)**				
Hypertension	258 (66.2)	22 (26.2)	73 (68.9)	163 (81.5)
Dyslipidemia	220 (56.4)	27 (32.1)	60 (56.6)	133 (66.5)
Chronic kidney disease	104 (26.7)	0 (0.0)	7 (6.6)	97 (48.5)
Osteoarthrosis	53 (13.6)	1 (1.2)	13 (12.3)	39 (19.5)
Obesity	49 (12.6)	3 (3.6)	11 (10.4)	35 (17.5)
Ischemic heart disease	36 (9.2)	0 (0.0)	9 (8.5)	27 (13.5)
Thyroid disease	37 (9.5)	1 (1.2)	7 (6.6)	29 (14.5)
Solid neoplasms	35 (9.0)	0 (0.0)	2 (1.9)	33 (16.5)
**Brain volumes (mL) at baseline, mean (SD)**				
TBTV	1057.9 (73.6)	1103.1 (57.2)	1069.8 (70.6)	1032.6 (70.8)
Hippocampal volume	7.5 (0.8)	7.9 (0.6)	7.6 (0.7)	7.3 (0.9)
Ventricular volume	39.3 (17.0)	31.3 (14.1)	38.1 (16.4)	43.4 (17.2)
WMH volume	5.7 (9.5)	2.9 (4.7)	4.7 (8.3)	7.4 (11.1)

*Note*: Missing data: 13 in *APOE* genotype. All volumes are adjusted for total intracranial volume.

Abbreviations: *APOE*: apolipoprotein E; MMSE: Mini‐Mental State Examination; TBTV: total brain tissue volume; WMH: white matter hyperintensities.

Compared to non‐multimorbid participants, individuals with mild multimorbidity, and even more so those with complex multimorbidity, were older, more likely to be women, and had a lower educational level. Among participants with mild multimorbidity the most common diseases were hypertension (68.9%), dyslipidemia (56.6%), and osteoarthrosis (12.3%); among participants with complex multimorbidity the most common diseases were hypertension (81.5%), dyslipidemia (66.5%), and chronic kidney disease (48.5%) (Table 1). In participants with mild and complex multimorbidity, the most impaired systems were the cardiocirculatory (73.6% and 87.5%, respectively) and the endocrine‐metabolic (67.0% and 83.5%), followed by the musculoskeletal system (21.7%) in individuals with mild and the genitourinary system (52.5%) in those with complex multimorbidity. The prevalence of the impairment of different systems in the overall population and by presence of mild/complex multimorbidity is reported in Table 2.

**TABLE 2:** Systemic impairment at baseline, in the overall study population and by presence of mild/complex multimorbidity.

System, *n* (%)	Overall (*N* = 390)	No multimorbidity (*N* = 84)	Mild multimorbidity (*N* = 106)	Complex multimorbidity (*N* = 200)
Diseases of the cardiocirculatory system	276 (70.8)	23 (27.4)	78 (73.6)	175 (87.5)
Diseases of the endocrine and metabolic system	270 (69.2)	32 (38.1)	71 (67.0)	167 (83.5)
Diseases of the genitourinary system	116 (29.7)	2 (2.4)	9 (8.5)	105 (52.5)
Musculoskeletal conditions	114 (29.2)	3 (3.6)	23 (21.7)	88 (44.0)
Diseases of the digestive system	54 (13.9)	2 (2.4)	5 (4.7)	47 (23.5)
Diseases of the respiratory system	49 (12.6)	3 (3.6)	7 (6.6)	39 (19.5)
Hematological and immunological conditions	47 (12.1)	2 (2.4)	2 (1.9)	43 (21.5)
Diseases of the eye	37 (9.5)	1 (1.2)	4 (3.8)	32 (16.0)
Cancers	35 (9.0)	0 (0.0)	2 (1.9)	33 (16.5)
Diseases of the ear, nose, and throat	22 (5.6)	1 (1.2)	2 (1.9)	19 (9.5)
Infectious diseases	2 (0.5)	0 (0.0)	0 (0.0)	2 (1.0)
Skin conditions	1 (0.3)	0 (0.0)	0 (0.0)	1 (0.5)

During the follow‐up (mean 5.9, SD 0.2 years), 30 individuals died; 115 participants dropped out before the first follow‐up assessment, and 23 dropped out after the first follow‐up assessment. The flow chart of study participation is reported in Figure S1. Participants who dropped out or died were older (mean difference 5.7 years, *p* < 0.001), had a lower educational level (32.1% vs 47.3% with university education, *p* = 0.003), and were more likely to exhibit complex multimorbidity at baseline (60.1% vs 44.6%, *p* = 0.001) than those who completed the follow‐up.

During the follow‐up, 12 participants developed dementia; among them, one did not exhibit multimorbidity at baseline, three had mild and eight had complex multimorbidity. Compared to participants who remained free from dementia, those who developed dementia during follow‐up experienced a faster reduction of TBTV (β*time −0.15, 95% confidence interval [CI] −0.27, −0.03) and a faster accumulation of WMH (β*time 0.35, 95% CI 0.21, 0.49); they also experienced a faster hippocampal shrinkage (β*time −0.14, 95% CI −0.29, 0.01) and ventricular enlargement (β*time 0.09, 95% CI −0.01, 0.18), even though statistically non‐significant.

In multiadjusted models, compared to non‐multimorbid participants, those with multimorbidity presented at baseline with a smaller TBTV (β −0.33, 95% CI −0.51, −0.14), and larger ventricular volume (β 0.27, 95% CI 0.03, 0.51). Over the 6‐year follow‐up, multimorbid participants experienced a steeper reduction in TBTV (additional annual change [β*time] −0.03, 95% CI −0.05, −0.01) and hippocampal volume (β*time −0.04, 95% CI −0.07, −0.01), in parallel with a faster ventricular enlargement (β*time 0.02, 95% CI 0.01, 0.04), as compared to the reference group (Table 3). They also exhibited a more severe WMH accumulation over time (β*time 0.03, 95% CI 0.01, 0.06) than non‐multimorbid individuals. Similarly, a higher number of chronic diseases, considered as a continuous variable, was associated with smaller TBTV and hippocampal volume and larger ventricular volume at baseline and with a steeper loss of TBTV and hippocampal volume and a faster increase in ventricular and WMH volume over the follow‐up (Table 3).

**TABLE 3:** Association between multimorbidity and brain volumes at baseline and over time.

	TBTV β (95% CI)	HV β (95% CI)	Ventricular volume β (95% CI)	WMH volume β (95% CI)
**Multimorbidity (baseline)**				
No multimorbidity	Ref.	Ref.	Ref.	Ref.
Multimorbidity	−0.33 (−0.51, −0.14)	−0.15 (−0.36, 0.07)	0.27 (0.03, 0.51)	0.12 (−0.14, 0.38)
**Multimorbidity (× time)**				
No multimorbidity	Ref.	Ref.	Ref.	Ref.
Multimorbidity	−0.03 (−0.05, −0.01)	−0.04 (−0.07, −0.01)	0.02 (0.01, 0.04)	0.03 (0.01, 0.06)
**Number of chronic diseases (baseline)**	−0.06 (−0.10, −0.02)	−0.06 (−0.10, −0.01)	0.07 (0.02, 0.12)	0.05 (−0.01, 0.11)
**Number of chronic diseases (× time)**	−0.01 (−0.01, 0.00)	−0.01 (−0.01, 0.00)	0.01 (0.00, 0.01)	0.01 (0.00, 0.01)

*Note*: Coefficients are derived from linear mixed models and are adjusted for age, sex, education and *APOE* genotype. All volumes are adjusted for total intracranial volume and converted into z‐scores.

Abbreviations: CI: confidence interval; HV, hippocampal volume; TBTV: total brain tissue volume; WMH: white matter hyperintensities.

**FIGURE 1:**
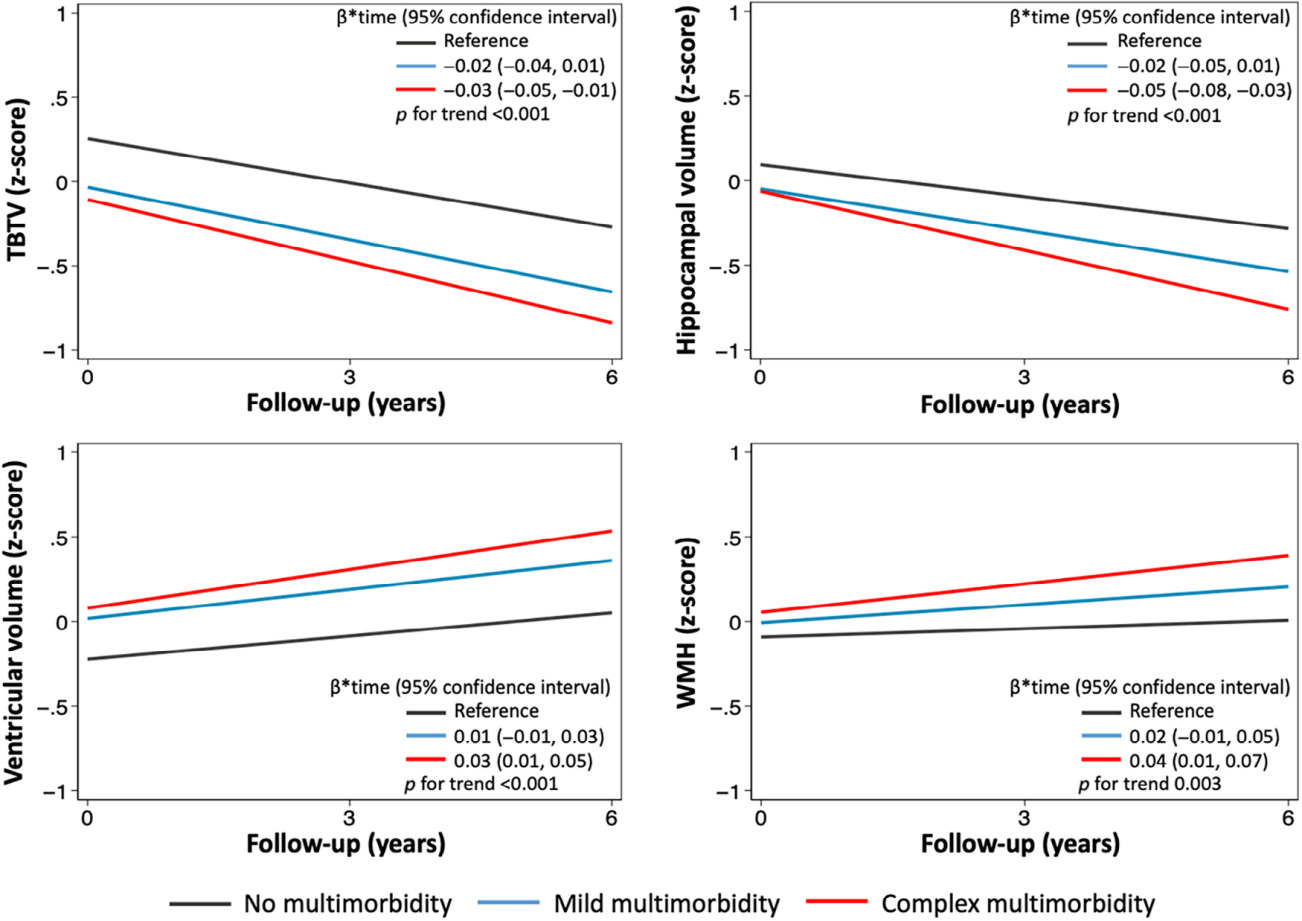
Trajectories of brain MRI changes over 6 years of follow‐up by presence of mild/complex multimorbidity. Linear mixed models were adjusted for age, sex, education and *APOE* genotype. All volumes are adjusted for total intracranial volume and converted into z‐scores. *APOE*, apolipoprotein E; MRI: magnetic resonance imaging; TBTV: total brain tissue volume; WMH: white matter hyperintensities.

Figure 1 and Table S2 show the rates of change of brain MRI volumes based on mild and complex multimorbidity, after controlling for potential confounders. At baseline, compared to individuals without multimorbidity, participants with complex multimorbidity had the smallest TBTV (β −0.36, 95% CI −0.57, −0.15) and the largest ventricular volume (β 0.30, 95% CI 0.04, 0.56), followed by those with mild multimorbidity. During follow‐up, individuals with complex multimorbidity experienced the most severe loss of TBTV (β*time −0.03, 95% CI −0.05, −0.01) and hippocampal volume (β*time −0.05, 95% CI −0.08, −0.03), followed by those with mild multimorbidity (β*time −0.02, 95% CI −0.04, 0.01 and −0.02, 95% CI −0.05, 0.01, for TBTV and hippocampal volume, respectively). Similarly, individuals with complex multimorbidity showed the greatest ventricular enlargement (β*time 0.03, 95% CI 0.01, 0.05), followed by those with mild multimorbidity (β*time 0.01, 95% CI −0.01, 0.03), and the most severe WMH accumulation (β*time 0.04, 95% CI 0.01, 0.07), followed by participants with mild multimorbidity (β*time 0.02, 95% CI −0.01, 0.05) over the 6‐year follow‐up.

## 4 DISCUSSION

The present study yielded the following key findings: individuals with multimorbidity—especially those with diseases involving multiple body systems—exhibited and accumulated higher loads of mixed brain pathological changes, encompassing both neurodegeneration and vascular pathology, relative to their non‐multimorbid peers. Specifically, the pathological changes observed in multimorbid individuals included a reduction in total brain tissue and hippocampal volumes, along with ventricular enlargement, and accumulation of WMH. These results were observed among dementia‐free individuals, suggesting that multimorbidity burden has an early detrimental effect on the brain.

The evidence on the link between multimorbidity and brain imaging is so far limited and mostly comes from cross‐sectional studies. Those studies found an association of multimorbidity with neuroimaging markers of neurodegeneration^13^, ^20^, ^21^, ^22^, ^23^ and cerebrovascular pathology.^21^, ^22^ For instance, Vassilaki et al.^20^ found that, in a population‐based sample of cognitively intact older adults, multimorbidity was linked with lower ^18^F‐fluorodeoxyglucose‐positron emission tomography metabolism in the Alzheimer's disease (AD) metaregion of interest and lower cortical thickness. In addition, the authors reported an association between the number of chronic diseases and cortical infarcts and smaller hippocampal volume, although no such association was observed with amyloid deposition. In a study on data from the UK Biobank,^22^ individuals with a higher number of chronic diseases had lower total brain tissue, grey matter, and hippocampal volume, and higher WMH load compared to those with low chronic disease burden. As opposed to those previous studies, we did not find significant differences in hippocampal and WMH volumes at baseline among individuals with different multimorbidity burden (except for the cross‐sectional association between a higher number of chronic diseases and lower hippocampal volume). This could be at least partially explained by the fact that we based our analyses on a selected dementia‐free population where we further excluded individuals with stroke, or any other neuropsychiatric disease, at baseline.

Besides quantifying the cross‐sectional association between multimorbidity and brain volumes, we here for the first time longitudinally explored the impact of multimorbidity on structural brain changes. We found that individuals with multimorbidity experienced a steeper reduction in total brain tissue volume, a faster ventricular enlargement, and a faster accumulation of WMH over time, as compared with their non‐multimorbid peers. We additionally explored the differential impact of multimorbidity on brain pathology based on the number of impaired body systems and found a dose‐response relationship between the complexity of multimorbidity and the rate of progression of brain atrophy and cerebrovascular load accumulation. Indeed, compared to non‐multimorbid participants, individuals who suffered from chronic diseases affecting multiple body systems (ie, complex multimorbidity) experienced the steepest trajectories of structural brain changes, followed by those with mild multimorbidity.

Studies using data from the UK Biobank observed that the association with smaller brain volumes was particularly strong for specific combinations of diseases (ie, metabolic and cardiometabolic multimorbidity).^15^, ^18^ Interestingly, we also observed that, within multimorbidity, it is not only a higher number of concomitant diseases but also the impairment of multiple body systems that exerts the most negative effects on the brain. In fact, the coexistence of multiple diseases affecting different body systems within the same individual seemed to determine a detrimental effect on the brain that goes beyond that of the mere sum of individual illnesses. These observations call for the need to focus on the whole clinical‐biological complexity of the organism when studying brain aging.

To allow comparability across coefficients and to investigate the potential pathophysiological mechanisms underlying the relationship between multimorbidity and structural brain changes, we standardized brain volumes. However, given the similarity of the coefficients, our results do not provide conclusive evidence that a specific brain structure is more susceptible to multimorbidity than others. Further research, involving larger sample sizes and other neuroimaging techniques, may help delve deeper into these aspects.

Several biological pathways may underlie the relationship between chronic disease burden and brain pathology, among others, vascular pathology, inflammation, and oxidative stress. Many chronic diseases, such as diabetes and hypertension, are well known to affect cerebral circulation, leading to the development of microvascular pathology (ie, microinfarcts, WMH) that may contribute to cognitive decline.^24^, ^25^ Vascular pathology and cerebral hypoperfusion have also been observed to contribute to grey matter atrophy.^26^, ^27^, ^28^ In addition, previous studies found an association between systemic inflammation and chronic disease burden,^29^ and increasing evidence also suggests that systemic inflammation may impact brain structure and cognition.^30^, ^31^ Another factor that may be involved in the relationship between multimorbidity, brain damage, and cognitive impairment is oxidative stress, which is known to increase with aging and has been linked to multiple chronic diseases,^32^ including AD and other dementias.^32^, ^33^

Our results add to existing evidence showing that somatic diseases contribute to dementia development and that both individual diseases^34^, ^35^, ^36^ as well as their combinations (ie, cardiometabolic, neuropsychiatric, sensory impairment/cancer multimorbidity)^6^, ^7^, ^9^, ^37^ negatively impact cognition. It is noteworthy that our findings come from a cognitively intact cohort, supporting the notion that multimorbidity exerts its negative impact on the brain even before a diagnosis of dementia. Taken as a whole, these results suggest that somatic diseases may be a target for dementia prevention strategies.

### 4.1 Strengths and limitations

Our findings come from a large, well‐established population‐based cohort with available repeated brain MRI measurements. A standardized protocol was followed for brain MRI acquisition and processing. At baseline, multimorbidity was operationalized based on comprehensive information on several chronic diseases collected through an extensive clinical evaluation. The following limitations should be also considered. First, SNAC‐K, and particularly SNAC‐K‐MRI, includes older adults living in central Stockholm who are relatively healthy and fit, have a high socioeconomic status, and are mainly born in Sweden. This might limit the generalizability of our results to other countries or populations. Furthermore, besides the brain MRI parameters considered in the current study, other neuroimaging markers (eg, microbleeds, amyloid deposition) are worthy of further investigation in this context, but they are not available in our population‐based dataset.

## 5 CONCLUSIONS

In a sample of older adults without dementia, a high disease burden, especially when multiple body systems are affected, accelerates the occurrence of mixed brain pathological changes, encompassing neurodegeneration and vascular pathology. These findings call for the need to further elucidate the mechanisms underlying the relationship between multimorbidity and dementia. Special attention should be given to older adults with a high chronic disease burden in clinical settings, and potential interventions should be explored to preserve brain health in individuals with multimorbidity.

## AUTHOR CONTRIBUTION

MV, DLV, and GG contributed to the conception and design of the study. MV conducted the statistical analyses. MV conducted the literature search. All authors contributed to the interpretation of the results. MV drafted the first version of the manuscript. All authors critically revised the manuscript for important intellectual content. All authors made a significant contribution to the research and the development of the manuscript and approved the final version for publication.

## CONFLICT OF INTEREST STATEMENT

The authors declare no conflicts of interest. Author disclosures are available in the supporting information.

## CONSENT STATEMENT

All participants provided written informed consent to participate at each study visit.

## Supporting information

Supporting Information

Supporting Information
