# Supplementary material for: Association of mild and complex multimorbidity with structural brain changes in older adults: A population‐based study
Source: Alzheimers Dement. 2024 Jan 3;20(3):1958–65. doi: 10.1002/alz.13614 (PMC10984455; doi:10.1002/alz.13614)
Supplement: Supplementary file 1 — Supporting Information [file ALZ-20-1958-s001.docx]

**Table S1.** List of chronic diseases by body systems

| **Cancers** | Hematological neoplasms |
| --- | --- |
|  | Solid neoplasms |
| **Diseases of the Cardio-circulatory System** | Atrial fibrillation |
|  | Bradycardias and conduction diseases |
|  | Cardiac valve diseases |
|  | Heart failure |
|  | Hypertension |
|  | Ischemic heart disease |
|  | Other cardiovascular disorders |
|  | Peripheral vascular disease |
|  | Venous and lymphatic diseases |
| **Diseases of the Digestive System** | Chronic liver diseases |
|  | Chronic pancreas, biliary tract and gallbladder diseases |
|  | Colitis and related disorders |
|  | Esophagus, stomach and duodenal disorders |
|  | Inflammatory bowel diseases |
|  | Other digestive diseases |
| **Diseases of Ear Nose and Throat** | Deafness and hearing loss |
|  | Ear, nose, throat diseases |
| **Diseases of the Endocrine-Metabolic System** | Diabetes |
|  | Dyslipidemia |
|  | Obesity |
|  | Other metabolic diseases |
|  | Thyroid disease |
| **Diseases of the Eye** | Blindness, visual impairment |
|  | Cataract and lens diseases |
|  | Glaucoma |
|  | Other eye diseases |
| **Diseases of the Genitourinary System** | Chronic kidney disease |
|  | Other genitourinary diseases |
|  | Prostate disorders |
| **Diseases of the Respiratory System** | Asthma |
|  | COPD, emphysema, and chronic bronchitis |
|  | Other respiratory diseases |
| **Hematological and Immunological conditions** | Allergy |
|  | Anemia |
|  | Autoimmune disorders |
|  | Blood and blood forming organ diseases |
| **Infectious Diseases** | Chronic infectious diseases |
| **Musculoskeletal conditions** | Dorsopathies |
|  | Inflammatory arthropathies |
|  | Osteoarthrosis and degenerative joint diseases |
|  | Osteoporosis |
|  | Other MSK and joint disorders |
| **Skin conditions** | Chronic ulcers of the skin |
|  | Other skin diseases |

**Table S2.** Association between mild/complex multimorbidity and brain volumes at baseline and over time.

|  | TBTV  β (95% CI) | HV  β (95% CI) | Ventricular volume  β (95%CI) | WMH volume  β (95%CI) |
| --- | --- | --- | --- | --- |
| **Multimorbidity (baseline)** |  |  |  |  |
| No multimorbidity | Ref. | Ref. | Ref. | Ref. |
| Mild multimorbidity | -0.29 ( -0.50, -0.07) | -0.14 (-0.39, 0.11) | 0.24 (-0.03, 0.51) | 0.09 (-0.21, 0.38) |
| Complex multimorbidity | -0.36 (-0.57, -0.15) | -0.16 (-0.39, 0.08) | 0.30 (0.04, 0.56) | 0.15 (-0.14, 0.43) |
| p for trend | 0.001 | 0.225 | 0.029 | 0.311 |
| **Multimorbidity** **(x time)** | |  |  |  |
| No multimorbidity | Ref. | Ref. | Ref. | Ref. |
| Mild multimorbidity | -0.02 (-0.04, 0.01) | -0.02 (-0.05, 0.01) | 0.01 (-0.01, 0.03) | 0.02 (-0.01, 0.05) |
| Complex multimorbidity | -0.03 (-0.05, -0.01) | -0.05 (-0.08, -0.03) | 0.03 (0.01, 0.05) | 0.04 (0.01, 0.07) |
| p for trend | <0.001 | <0.001 | <0.001 | 0.003 |
|  |  |  |  |  |

Coefficients are derived from linear mixed models and are adjusted for age, sex, education and *APOE* genotype.

All volumes are adjusted for total intracranial volume and converted into z-scores.

Abbreviations: CI: confidence interval; TBTV: total brain tissue volume; WMH: white matter hyperintensities.

**Figure S1.** Flow chart of study participation over 6 years


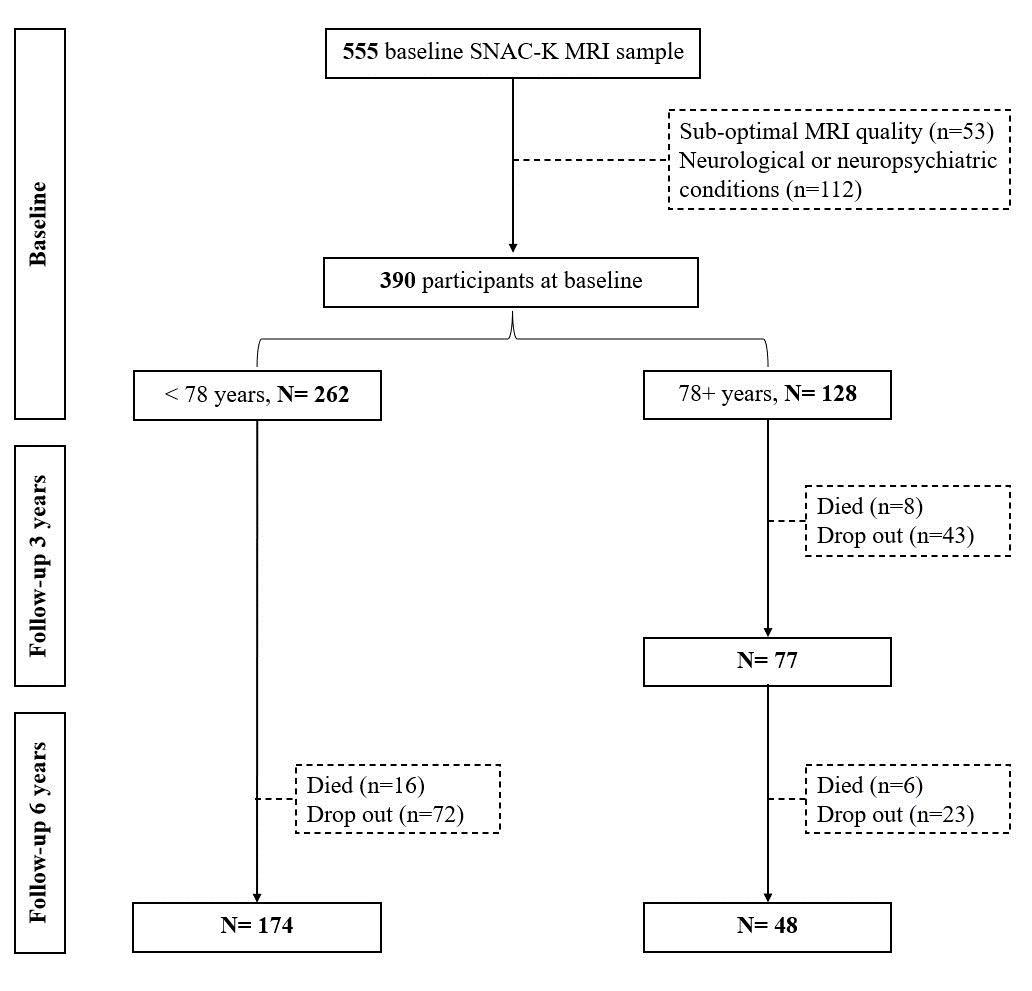


**Supplementary methods.** Magnetic resonance imaging (MRI) protocol and imaging acquisition

Participants underwent MRI scans with a 1.5T MRI scanner (Philips Intera, The Netherlands). The protocol included: 1) an axial 3D T1-weighted fast field echo (repetition time [TR] 15 ms, echo time [TE] 7 ms, flip angle [FA] 15°, field of view [FOV] 240, 128 slices with slice thickness 1.5 mm and in-plane resolution 0.94 × 0.94 mm, no gap, matrix 256 × 256), and 2) an axial turbo fluid-attenuated inversion recovery sequence (FLAIR; TR 6000 ms, TE 100 ms, inversion time 1900 ms, FA 90°, echo train length 21, FOV 230, 22 slices with slice thickness 5 mm and in-plane resolution 0.90 × 0.90 mm, gap 1 mm, matrix 256 × 256).

Grey matter, white matter, and cerebrospinal fluid volumes were derived after segmentation of the T1-weighted images in SPM12 (Statistical Parametric Mapping, http://www.fil.ion.ucl.ac.uk/spm/, Wellcome Trust Centre for Neuroimaging, FIL, London, UK), implemented in Matlab 10 (The Mathworks Inc., MA, US), using the improved unified segmentation algorithm^1^ that employs an extended set of tissue-probability maps. The “light cleanup” option was used to further remove odd voxels from the images. All segmentations were inspected by a neuroimaging expert (G.K.). Hippocampal volume was measured using automated segmentation of the T1-weighted images performed with the Freesurfer 5.1 image-analysis suite (http://surfer.nmr.mgh.harvard.edu/)^2^. To compute white matter hyperintensities volume, G.K. manually drew on FLAIR images white matter hyperintensities that were further interpolated on the corresponding T1 images to compensate for the gap between slices in FLAIR^3^ (intra-rater reliability was high >0.987).

**References**

1. Ashburner, J. & Friston, K. J. Unified segmentation. NeuroImage 26, 839–851 (2005).

2. Fischl, B. et al. Whole brain segmentation: automated labeling of neuroanatomical structures in the human brain. Neuron 33, 341–355 (2002).

3. Köhncke, Y. et al. Three-year changes in leisure activities are associated with concurrent changes in white matter microstructure and perceptual speed in individuals aged 80 years and older. Neurobiol. Aging 41, 173–186 (2016).
